# Lung tissue bioenergetics and caspase activity in rodents

**DOI:** 10.1186/1756-0500-6-12

**Published:** 2013-01-12

**Authors:** Ahmed R Alsuwaidi, Mohammed T Alsamri, Ali S Alfazari, Saeeda Almarzooqi, Alia Albawardi, Aws R Othman, Thachillath Pramathan, Stacey M Hartwig, Steven M Varga, Abdul-Kader Souid

**Affiliations:** 1 Department of Pediatrics, United Arab Emirates University, P.O. Box 17666, Al Ain, UAE; 2 Department of Medicine, United Arab Emirates University, P.O. Box 17666, Al Ain, UAE; 3 Department of Pathology, United Arab Emirates University, P.O. Box 17666, Al Ain, UAE; 4 Department of Microbiology, University of Iowa, Iowa City, IA, 52242, USA; 5 Department of Pathology and Interdisciplinary Graduate Program in Immunology, University of Iowa, Iowa City, IA, 52242, USA

**Keywords:** *In vitro*, Cytotoxicity, Apoptosis

## Abstract

**Background:**

This study aimed to establish a suitable *in vitro* system for investigating effects of respiratory pathogens and toxins on lung tissue bioenergetics (cellular respiration and ATP content) and caspase activity. Wistar rats and C57Bl/6 mice were anesthetized by sevoflurane inhalation. Lung fragments were then collected and incubated at 37°C in a continuously gassed (with 95% O_2_:5% CO_2_) Minimal Essential Medium (MEM) or Krebs-Henseleit buffer. Phosphorescence O_2_ analyzer that measured dissolved O_2_ concentration as a function of time was used to monitor the rate of cellular mitochondrial O_2_ consumption. Cellular ATP content was measured using the luciferin/luciferase system. The caspase-3 substrate *N*-acetyl-asp-glu-val-asp-7-amino-4-methylcoumarin (Ac-DEVD-AMC) was used to monitor intracellular caspase activity; cleaved AMC moieties (reflecting caspase activity) were separated on HPLC and detected by fluorescence. Lung histology and immunostaining with anti-cleaved caspase-3 antibody were also performed.

**Results:**

For Wistar rats, the values of *k*_*c*_ and ATP for 0 < *t* ≤ 7 h (mean ± SD) were 0.15 ± 0.02 μM O_2_ min^-1^ mg^-1^ (n = 18, coefficient of variation, Cv = 13%) and 131 ± 69 pmol mg^-1^ (n = 16, Cv = 53%), respectively. The AMC peak areas remained relatively small despite a ~5-fold rise over 6 h. Good tissue preservation was evident despite time-dependent increases in apoptotic cells. Lung tissue bioenergetics, caspase activity and structure were deleterious in unoxygenated or intermittently oxygenated solutions. Incubating lung tissue in O_2_ depleted MEM for 30 min or anesthesia by urethane had no effect on lung bioenergetics, but produced higher caspase activity.

**Conclusions:**

Lung tissue bioenergetics and structure could be maintained *in vitro* in oxygenated buffer for several hours and, thus, used as biomarkers for investigating respiratory pathogens or toxins.

## Background

*In vitro* systems for studying tissue mitochondrial O_2_ consumption (cellular respiration) and morphology in BALB/c mice are recently described [1-3]. These parameters, however, are insufficient for fully assessing cellular viability. For example, cells may respire (consume O_2_) at a higher rate due to uncoupling oxidative phosphorylation (short-circuiting the inner mitochondrial membrane), an event that results from increased caspase activities [4]. Therefore, measuring cellular ATP and caspase activity are essential biomarkers for a more comprehensive assessment of tissues *in vitro*.

Lung toxicity is a major health concern in the drug development. The US Food and Drug Administration (FDA) has issued several post-marketing drug recalls due to adverse events related to mitochondrial dysfunctions [5]. Thus, developing *in vitro* systems to assess adverse effects of drugs on lung tissue bioenergetics is important. Drug-induced mitochondrial disturbances have been investigated using different methods, including fluorescence-based oxygen sensitive probes [6-8].

In this study, the previously described method [1-3,7] is expanded to include measurements of lung tissue ATP content and caspase activity in Wistar rats and C57Bl/6 mice. The work aimed to establish an *in vitro* system suitable for investigating respiratory pathogens and toxins. The impacts of experimental conditions (e.g., anesthetic agents, incubation solutions, and oxygenation) are also addressed.

## Methods

Pd(II) complex of *meso*-tetra-(4-sulfonatophenyl)-tetrabenzoporphyrin **(**Pd phosphor**)** was purchased from Porphyrin Products (Logan, UT). A lyophilized powder of caspase inhibitor I [*N*-benzyloxycarbonyl-val-ala-asp(O-methyl)-fluoromethylketone; zVAD-fmk; *m.w.* = 467.5; pan-caspase inhibitor] was purchased from Calbiochem (La Jolla, CA). Ac-DEVD-AMC (*N*-acetyl-asp-glu-val-asp-7-amino-4-methylcoumarin; *m.w.* = 675.64; caspase-3 substrate) was purchased from Axxora LLC (San Diego, CA). Minimum Essential Medium (MEM Alpha Modification) was purchased from Gibco (labeled here as MEM). Dactinomycin (actinomycin D, MW ≈ 1255) was purchased from Merck (Whitehouse Station, NJ). Glucose (anhydrous) and remaining reagents were purchased from Sigma-Aldrich (St. Louis, MO).

zVAD-fmk solution (2.14 mM) was made by dissolving 1.0 mg in 1.0 mL dimethyl sulfoxide and stored at -20°C in small aliquots. Ac-DEVD-AMC solution (7.4 mM) was made by dissolving 5.0 mg in 1.0 mL dimethyl sulfoxide and stored at -20°C. Pd phosphor solution (2.5 mg/ml = 2 mM) was prepared in dH_2_O and stored at -20°C in small aliquots. Dactinomycin solution was made fresh in dH_2_O; its concentration was determined by absorbance at 440 nm, using an extinction coefficient of 24,450 M^-1^ · cm^-1^. Sodium cyanide (NaCN) solution (1.0 M) was prepared in dH_2_O; the *p*H was adjusted to ~7.0 with 12N HCl and stored at -20°C. Glucose oxidase (10 mg/mL) was dissolved in dH_2_O and stored at -20°C. Krebs-Henseleit (KH) buffer (115 mM NaCl, 25 mM NaHCO_3_, 1.23 mM NaH_2_PO_4_, 1.2 mM Na_2_SO_4_, 5.9 mM KCL, 1.25 mM CaCl_2_, 1.18 mM MgCl_2_ and 10 mM glucose, *p*H ~7.4) was made fresh.

### Animals

Wistar rats (weighing about 215 grams) and C57Bl/6 mice (weighing about 32.5 grams) and BALB/c mice (Additional file 1: Table S2) used in this study were maintained at the animal facility that was in compliance with NIH guidelines (http://grants.nih.gov/grants/olaw/references/phspol.htm). All animals were housed in rooms maintained at 22°C with ~60% relative humidity and a 12-hr light/dark cycle. They had *ad libitum* access to standard rodent chow and filtered water. The study was approved by the Animal Research Ethics Committee for care and use of laboratory animals at the College of Medicine and Health Sciences, UAE University.

### Lung tissue

The animals were anesthetized by sevoflurane inhalation (100 μL per 10 g) and sacrificed as outlined [2,3]. Lung specimens were *immediately* immersed in continuously gassed with 95% O_2_:5% CO_2_ ice-cold MEM or KH buffer. For O_2_ measurements, specimens were placed in 1.0 mL MEM or KH buffer containing 0.5% fat-free bovine albumin and 3 μM Pd phosphor. Specimens were also processed for histology and measurements of caspase activity and ATP content as described below.

Specimens were fixed in 10% buffered formalin solution and embedded in paraffin wax blocks. Sections of the fixed lung pieces (of 3 μm thickness) were stained with haematoxylin and eosin (H&E) and examined under a light microscope.

Staining for apoptosis was performed using avidin-biotin immunoperoxidase method that detects activated caspase-3 (Cell Signaling Technology, Boston, MA). The procedure was performed on 3-μm paraffin sections using rabbit anti-cleaved caspase-3 antibody. Positive and negative control sections for apoptosis were used.

### Intracellular caspase activity

Lung fragments (about 20 mg each) were collected from Wistar rats and C57Bl/6 mice as described [2]. The samples were incubated *in vitro* at 37°C in 50 mL KH buffer or MEM (continuously gassed with 95% O_2_:5% CO_2_) for up to 6 h. At specific time points, samples were incubated in oxygenated KH buffer or MEM with 32 μM zVAD-fmk or 15 μL DMSO for 20 min (f/v = 1.0 mL). Ac-DEVD-AMC (37 μM) was then added and the incubation continued for additional 20 min. At the end of incubation, the tissue was disrupted by vigorous homogenization for 2 min, sonication for 3 min and 10 passages through a 27-G needle. This disruption procedure quenched the Ac-DEVD-AMC cleavage reaction due to dilution. The supernatants were collected by centrifugation (~16,300*g* for 90 min) through Microcentrifuge Filter (nominal molecular weight limit = 10,000 Dalton, Sigma^©^), separated on HPLC, and analyzed for the fluorogenic AMC moiety.

### HPLC

The analysis was performed on a Waters reversed-phase HPLC system, which consisted of a manual injector, a pump and a fluorescent detector. The excitation wavelength was 380 nm and the emission wavelength 460 nm. Solvents A and B were HPLC-grade methanol:dH_2_O 1:1 (isocratic). The column, 4.6 × 250 mm Beckman Ultrasphere IP column, was operated at 25°C at 1.0 ml/min. The run time was 10 min and the injection volume was 20 μL.

### Oxygen measurements

A phosphorescence oxygen analyzer was used to monitor O_2_ consumption by lung specimens [2,3]. Briefly, O_2_ detection was performed with the aid of Pd phosphor (absorption maximum at 625 nm and phosphorescence maximum at 800 nm). Samples were exposed to light flashes (600 per min) from a pulsed light-emitting diode array with peak output at 625 nm (OTL630A-5-10-66-E, Opto Technology, Inc., Wheeling, IL). Emitted phosphorescent light was detected by a Hamamatsu photomultiplier tube after passing through an interference filter centered at 800 nm. The amplified phosphorescence decay was digitized at 1.0 MHz by a 20-MHz A/D converter (Computer Boards, Inc., Mansfield, MA).

A program was developed using Microsoft Visual Basic 6, Microsoft Access Database 2007, and Universal Library components (Universal Library for Measurements Computing Devices; http://www.mccdaq.com/daq-software/universal-library.aspx). It allowed direct reading from the PCI-DAS 4020/12 I/O Board (PCI-DAS 4020/12 I/O Board; http://www.mccdaq.com/pci-data-acquisition/PCI-DAS4020-12.aspx). The pulse detection was accomplished by searching for 10 phosphorescence intensities >1.0 volt (by default). Peak detection was accomplished by searching for the highest 10 data points of a pulse and choosing the data point closest to the pulse decay curve [7].

The phosphorescence decay rate (1/τ) was characterized by a single exponential; I = Ae^-*t*/τ^, where I = Pd phosphor phosphorescence intensity. The values of 1/τ were linear with dissolved O_2_: 1/τ = 1/τ^o^ + *k*_*q*_[O_2_, where 1/τ = the phosphorescence decay rate in the presence of O_2_, 1/τ^o^ = the phosphorescence decay rate in the absence of O_2_, and *k*_q_ = the second-order O_2_ quenching rate constant in s^-1^ · μM^-1^[8].

Lung tissue respiration was measured at 37°C in 1-mL sealed vials. Mixing was with the aid of parylene-coated stirring bars. In vials sealed from air, [O_2_] decreased linearly with time, indicating the kinetics of mitochondrial O_2_ consumption was zero-order. The rate of respiration (*k*, in μM O_2_ min^-1^) was thus the negative of the slope d[O_2_]/d*t*. Sodium cyanide (NaCN) inhibited respiration, confirming O_2_ was being consumed in the mitochondrial respiratory chain.

The calibration reaction contained PBS with 3 μM Pd phosphor, 0.5% fat-free albumin, 50 μg/mL glucose oxidase and various concentrations of β-glucose [2]. [O_2_ was calculated using, 1/τ = 1/τ^o^ + *k*_*q*_[O_2_[8].

### Cellular ATP contents

Lung tissue fragments were homogenized in 0.5 mL of ice-cold 2% trichloroacetic acid for 2 min. The supernatants were collected by centrifugation (1000x*g* at 4°C for 5 min) and stored at -20°C until analysis. Immediately before ATP measurement, the samples were thawed and neutralized with 0.5 mL 100 mM Tris-acetate, 2 mM EDTA (final *p*H, 7.75). ATP concentration was determined using the Enliten ATP Assay System (Bioluminescence Detection Kit, Promega, Madison, WI). Briefly, 2.5 μL of the supernatant was added to 25 μL of the luciferin/luciferase reagent. The luminescence intensity was then measured at 25°C using Glomax Luminometer (Promega, Madison, WI). The ATP standard curve ranged from 10 pM to 100 nM (*R*^*2*^ >0.9999).

## Results

**Figure 1:**
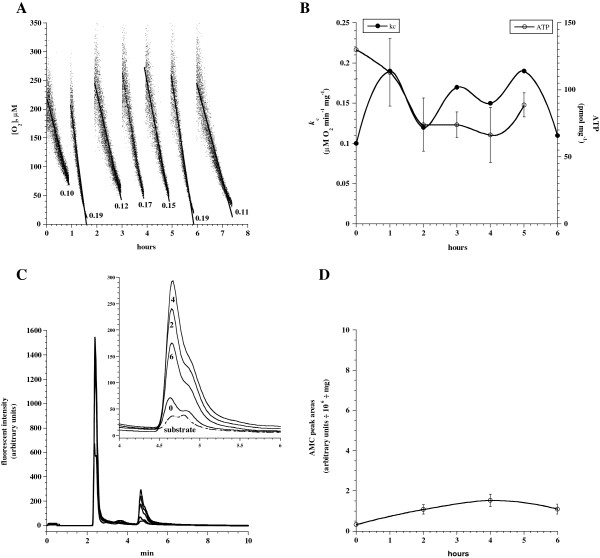
**Wistar rat lung tissue respiration, ATP content and caspase activity in oxygenated MEM.** Lung fragments were incubated at 37°C in 50 mL MEM (continuously gassed with 95% O_2_: 5% CO_2_) for up to ~7 h. At indicated time periods, samples were removed from the incubation medium and processed for measurements of O_2_ consumption, ATP content and caspase activity. **Panel A**: Runs of cellular mitochondrial O_2_ consumption are shown; *t* = 0 corresponds to animal sacrifice. The rate of respiration (*k,* μM O_2_ min^-1^) was set as the negative of the slope of [O_2_] *vs. t*. The values of *k*_*c*_ (μM O_2_ min^-1^ mg^-1^) are shown at the bottom of each run. **Panel B**: The values of *k*_*c*_ and ATP are plotted as a function of incubation time. **Panel C**: HPLC runs of caspase activity at 0, 2, 4 and 6 h of incubation. The retention time (*R*_t_) for Ac-DEVD-AMC was ~2.5 min and AMC ~4.8 min (insert, reflecting caspase activity). The substrate run (dashed line) was sample incubated at 37°C without lung specimen for 6 h. **Panel D**: AMC peak areas are shown as a function of incubation period.

**Figure 2:**
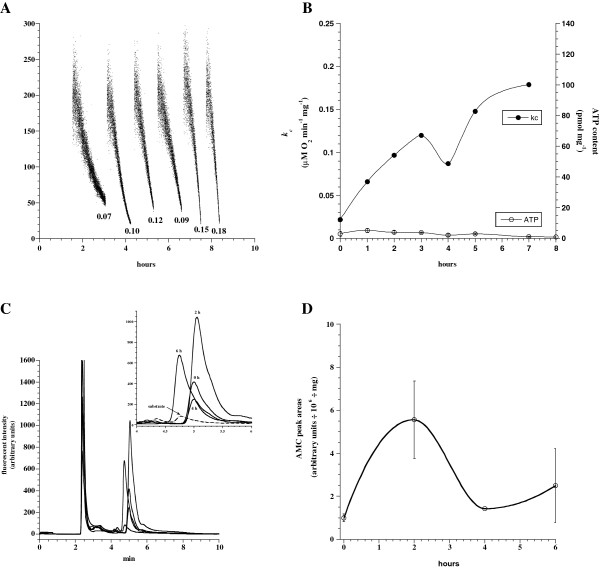
**Wistar rat lung tissue respiration, ATP content and caspase activity in unoxygenated KH buffer.** Lung fragments were incubated at 37°C in 50 mL KH buffer for up to ~8 h. At indicated time periods, samples were removed from the incubation medium and processed for measurements of O_2_ consumption, ATP content and caspase activity. **Panel A**: Runs of cellular mitochondrial O_2_ consumption are shown; *t* = 0 corresponds to animal sacrifice. The values of *k*_*c*_ (μM O_2_ min^-1^ mg^-1^) are shown at the bottom of each run. **Panel B**: The values of *k*_*c*_ and ATP are plotted as a function of incubation time. **Panel C**: HPLC runs of caspase activity at 0, 2, 4 and 6 h of incubation. The *R*_t_ for Ac-DEVD-AMC was ~2.5 min and AMC ~4.8 min (insert). The substrate run (dashed line) was sample incubated at 37°C without lung specimen for 6 h. **Panel D**: AMC peak areas are shown as a function of incubation period.

Figure 1 shows Wistar rat lung tissue respiration, ATP content and caspase activity in 50 mL MEM that was continuously gassed with 95% O_2_: 5% CO_2_. Lung fragments were incubated at 37°C for up to ~7 h. At indicated time periods, samples were removed from the incubation medium and processed for measurement of cellular mitochondrial O_2_ consumption (expressed as, *k*_*c*_ in μM O_2_ min^-1^ mg^-1^), ATP content (pmol mg^-1^) and caspase activity (AMC peak areas in arbitrary units mg^-1^). Figure 1A shows the values (mean ± SD) of *k*_*c*_ for 0 ≤ *t* ≤ 7 h were 0.147 ± 0.038 (n = 7, Cv = 26%). The corresponding values of cellular ATP (Figure 1B, done in triplicates) for 0 ≤ *t* ≤ 6 h was 91.0 ± 25.3 pmol mg^-1^ (n = 6, Cv = 28%). Figure 1C–D show the AMC peak areas (arbitrary units mg^-1^ ÷10^6^ performed in duplicates, reflecting caspase activity) for 0 ≤ *t* ≤ 6 h were 1.0 ± 0.5 (n = 8). These AMC peak areas were relatively small (compared to Figure 2D) despite the 3 to 5 fold increments over 6 h. The AMC peak area for Ac-DEVD-AMC incubated without lung specimen (substrate alone) at hour 6 was also relatively small (Figure 1C–D). Thus, this preparation resulted in a stable cellular bioenergetics (respiration and ATP content) with minimal caspase activity for at least 6 h.

The need for continuous gassing with O_2_:CO_2_ was then investigated. Figure 2A–D show Wistar rat lung tissue respiration, ATP content and caspase activity in KH buffer without gassing. The values of *k*_*c*_ (1.5 ≤ *t* ≤ 8 h) were 0.118 ± 0.041 (n = 6; Cv = 35%), which were lower than those in Figure 1A (*p* = 0.181). The values of cellular ATP (Figure 2B, done in triplicates for 0 ≤ *t* ≤ 8 h) was 2.9 ±1.4 pmol mg^-1^ (n = 8, Cv = 48%), which were significantly less (*p* = 0.001) than in Figure 1B. Figure 2D shows the AMC moiety was highest at 2 h (area = 5.6 ± 1.8), while the AMC peak area for Ac-DEVD-AMC incubated without lung specimen (substrate alone) for 6 h was relatively small. Thus, continuous tissue oxygenation was necessary for maintaining cellular bioenergetics and preventing caspase activation. Furthermore, the incubation in room air (21% O_2_) invoked deleterious effects on cellular bioenergetics with increased caspase activity.

The same experiment was repeated in room air supplemented with 5% CO_2_. Briefly, lung fragments from a Wistar rat were incubated at 37°C in 50 mL MEM in a CO_2_ tissue culture incubator. Samples were removed from the incubation medium at 0, 3 and 6 h and processed for measurements of O_2_ consumption, ATP content and caspase activity. The values of *k*_*c*_ (μM O_2_ min^-1^ mg^-1^) increased with time and the values of ATP (pmol mg^-1^) decreased with time, confirming uncoupling of oxidative phosphorylation (Additional file 1: Figure S2). Thus, the incubation in 21% O_2_:5% CO_2_ also invoked deleterious effects on cellular bioenergetics.

**Figure 3:**
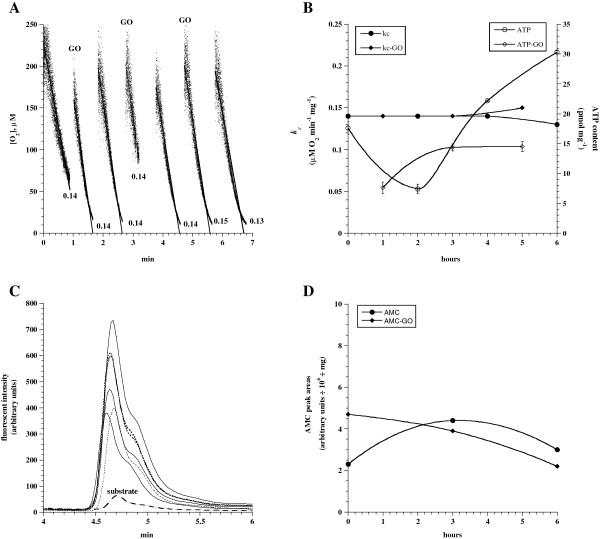
**Wistar rat lung tissue respiration, ATP content and caspase activity with and without 30 min prior incubation with glucose oxidase.** Lung fragments were incubated at 37°C in 2.0 mL MEM with and without 200 μg glucose oxidase (which totally depleted O_2_ from the solution) for 30 min. The samples were then rinsed with MEM and incubated at 37°C in 50 mL MEM (continuously gassed with 95% O_2_: 5% CO_2_) for up to ~7 h. At indicated time periods, samples were removed from the incubation medium and processed for measurements of O_2_ consumption, ATP content and caspase activity. **Panel A**: Runs of cellular mitochondrial O_2_ consumption are shown; *t* = 0 corresponds to animal sacrifice. The values of *k*_*c*_ (μM O_2_ min^-1^ mg^-1^) are shown at the bottom of each run. **Panel B**: The values of *k*_*c*_ and ATP are plotted as a function of incubation time. **Panel C**: HPLC runs of caspase activity at 0, 3 and 6 h of incubation, showing only the AMC peak at ~4.8 min (solid lines, with GO; dotted lines, with GO). The substrate run (dashed line) was sample incubated without lung specimen for 6 h. **Panel D**: AMC peak areas are shown as a function of incubation period.

To further investigate the impaired bioenergetics and caspase activation observed in Figure 2, Wistar rat lung tissue was incubated in O_2_ depleted MEM for 30 min. Briefly lung fragments were incubated at 37°C in 2.0 mL MEM with and without 200 μg glucose oxidase (which totally depleted O_2_ from the solution) for 30 min. The samples were then rinsed with MEM and incubated at 37°C in 50 mL MEM (continuously gassed with 95% O_2_: 5% CO_2_) for up to ~7 h. At indicated time periods, samples were removed from the incubation medium and processed for measurements of O_2_ consumption, ATP content and caspase activity. As shown in Figure 3A, the values of *k*_*c*_ with and without pre-incubation with glucose oxidase were the same 0.14 ± 0.00 (n = 3 and 4, respectively). The values of cellular ATP (done in triplicates) without treatment with glucose oxidase were 19.4 ± 9.6 pmol mg^-1^ (n = 4, Cv = 49%) and with glucose oxidase 12.1 ± 3.9 pmol mg^-1^ (n = 3, Cv = 32%), *p* = 0.400 (Figure 3B). Although, the AMC peak with glucose oxidase was relatively higher immediately post tissue collection (Figure 3C–D), its value did not significantly increase with time indicating no further caspase activation. The significance of background caspase activity at min zero (lung specimens immediately immersed in the Ac-DEVD-AMC reaction for 20 min) was unclear. The AMC peak area for Ac-DEVD-AMC incubated without lung specimen for 6 h was relatively small. Thus, the 30-min O_2_ depletion had no measurable effects on cellular bioenergetics and caspase activity.

**Figure 4:**
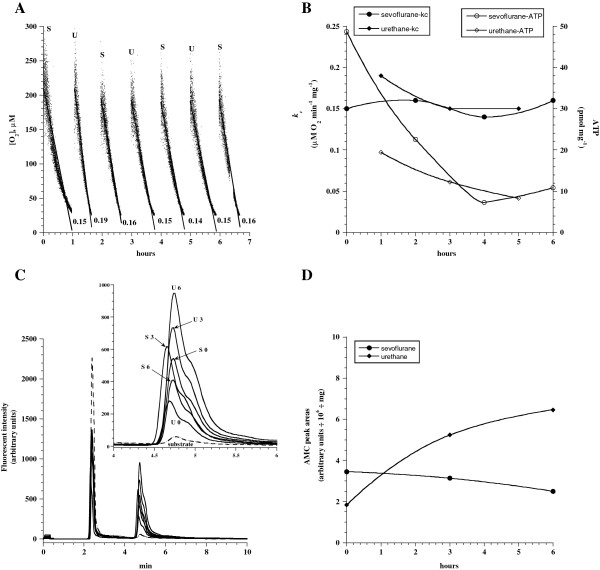
**Lung tissue respiration, ATP content and caspase activity in Wistar rats anesthetized with sevoflurane (S) or urethane (U).** Lung fragments were incubated at 37°C in 50 mL MEM (continuously gassed with 95% O_2_: 5% CO_2_) for up to ~7 h. At indicated time periods, samples were removed from the incubation medium and processed for measurements of O_2_ consumption, ATP content and caspase activity. **Panel A**: Runs of cellular mitochondrial O_2_ consumption are shown; *t* = 0 corresponds to animal sacrifice. The values of *k*_*c*_ (μM O_2_ min^-1^ mg^-1^) are shown at the bottom of each run. **Panel B**: The values of *k*_*c*_ and ATP are plotted as a function of incubation time. **Panel C**: HPLC runs of caspase activity at 0, 3 and 6 h of incubation. The *R*_t_ for Ac-DEVD-AMC was ~2.5 min and AMC ~4.8 min (insert). The substrate run (dashed line) was without lung specimen at 6 h. **Panel D**: AMC peak areas are shown as a function of incubation period.

The effects of the anesthetic agent sevoflurane *vs.* urethane were then tested. Lung fragments were incubated at 37°C in 50 mL MEM (continuously gassed with 95% O_2_: 5% CO_2_) for up to ~7 h. The values of *k*_*c*_ for sevoflurane anesthesia were 0.15 ± 0.01 (n = 4; Cv = 7%) and for urethane 0.16 ± 0.02 (n = 3; Cv = 13%), *p* = 0.857 (Figure 4A). The values of cellular ATP (Figure 4B, done in triplicates) for sevoflurane was 22.4 ± 18.7 pmol mg^-1^ (n = 4, Cv = 83%) and for urethane 13.3 ± 5.6 pmol mg^-1^ (n = 3, Cv = 42%), *p* = 0.857 (Figure 4B). The AMC moiety was stable with sevoflurane anesthesia over 6 h (3.0 ± 0.5, n = 3), but progressively increased with time for urethane anesthesia (4.5 ± 2.4, n = 3; *p* = 0.700), Figure 4C–D. The AMC peak area for Ac-DEVD-AMC incubated without lung specimen for 6 h was relatively small. Therefore, sevoflurane was the preferred anesthetic agent.

**Figure 5:**
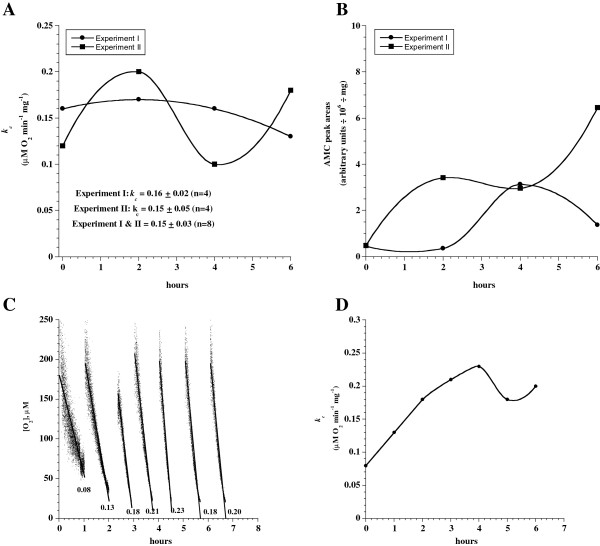
**C57Bl/6 mouse lung tissue respiration and caspase activity. Panels A–B**, in 2 independent experiments, lung fragments were incubated at 37°C in 50 mL KH buffer gassed for 60 sec with 95% O_2_ : 5% CO_2_ every h. At indicated time periods, samples were removed from the incubation medium and processed for O_2_ consumption and caspase activity. The values of *k*_*c*_ (**Panel A**) and AMC peak area (**Panel B**) are plotted as a function of incubation time. **Panel C–D**, respiration of lung fragments that were incubated at 37°C in 50 mL MEM continuously gassed for 60 sec with 95% O_2_:5% CO_2_.

Figure 5 shows two independent experiments of C57Bl/6 mouse lung tissue respiration and caspase activity in KH buffer (intermittently 60 sec every h) gassed with 95% O_2_: 5% CO_2_. The values of *k*_*c*_ for both experiments were 0.15 ± 0.03 (Figure 5A). The AMC peak was increased with time in both experiments (Figure 5B). In Figure 5C–D, the same experiment was repeated in MEM that was continuously gassed with 95% O_2_:5% CO_2_. The values of *k*_*c*_ for both experiments were 0.17 ± 0.05 (n = 7, Cv = 29%).

**Figure 6:**
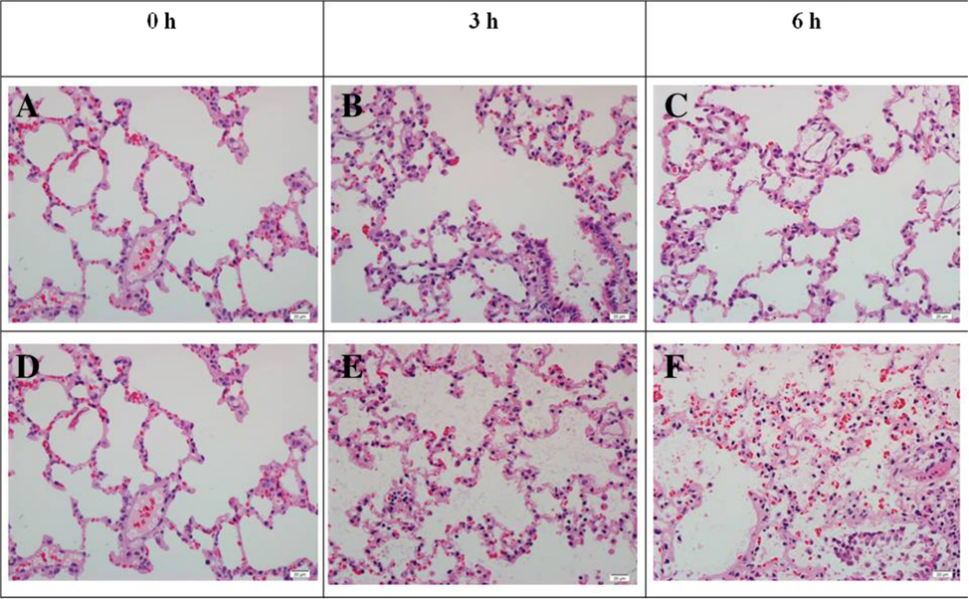
**Lung tissue histology.** Lung fragments from a Wistar rat were incubated at 37°C in 50 mL MEM continuously gassed with 95% O_2_:5% CO_2_ (**Panels B–C**) or kept without gassing (**Panels E–F**). The tissues were then processed for H&E staining at 0, 3, and 6 h. For **Panel A–D**, the lung specimen was fixed immediately after tissue collection. The pulmonary parenchyma was better preserved in oxygenated sample over 6 h compared to unoxygenated sample, which demonstrated signs of tissue death overtime characterized by collapse of alveolar walls with sloughing of bronchiolar epithelium, loss of pneumatocytes, extravasation of red blood cells and serum fluid into alveolar spaces. (Hematoxylin and eosin, H&E, 40x).

Light microscopy of lung tissue from a Wistar rat is shown in Figure 6. The specimens were incubated at 37°C in MEM continuously gassed with 95% O_2_:5% CO_2_ or kept at 37°C without gassing for 0, 3, and 6 h. At 0 h (Figure 6A), there is good tissue preservation with unremarkable pulmonary parenchyma composed of delicate thin alveolar walls lined by pneumatocytes. In oxygenated MEM at 3 h (Figure 6B), the pulmonary parenchyma is well preserved and composed of delicate thin alveolar wall lined by unremarkable pneumatocytes and a preserved bronchiolar epithelium; a few alveolar macrophages were noted. Tissue architecture is partially preserved at 6 h, with foci of alveolar edema, cytoplasmic disintegration of the alveolar lining epithelium and a few alveolar macrophages (Figure 6C). In unoxygenated MEM, tissue architecture was still preserved at 3 h, but with appearance of alveolar macrophages and early cytoplasmic disintegration of the alveolar lining epithelium (Figure 6E). Tissue preservation was poor at 6 h, showing collapse of alveolar walls, sloughing of bronchiolar epithelium and extravasation of red blood cells and fluid into alveolar spaces (Figure 6F).

**Figure 7:**
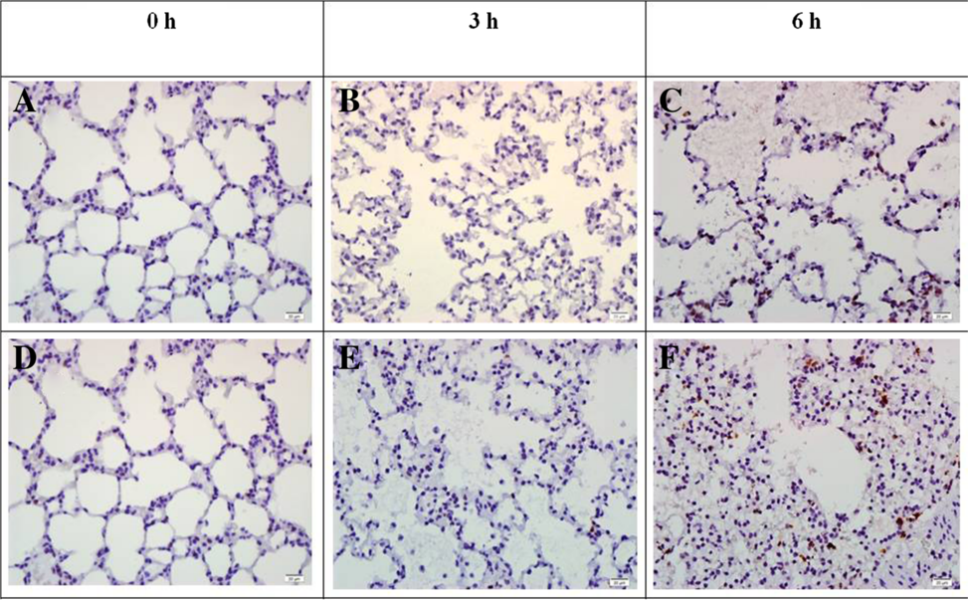
**Lung tissue caspase-3 immunostaining.** Lung fragments from a Wistar rat were incubated at 37°C in 50 mL MEM continuously gassed with 95% O_2_:5% CO_2_ (**Panels B–C**) or kept without gassing (**Panels E–F**). The tissues were then stained with rabbit anti-cleaved caspase-3 antibody at 0, 3, and 6 h. For **Panel A–D**, the lung specimen was fixed immediately after tissue collection. Please note that the H&E (Figure 6) and caspase-3 immunostain (Figure 7) pictures correspond to the same areas. There was an increase in percentage of cells in apoptosis with time (brown staining cells) in both experimental conditions. (Immunoperoxidase, 40x).

Lung tissue immunostaining with rabbit anti-cleaved caspase-3 antibody is shown in Figure 7; the pictures correspond to the same areas in Figure 6. The apoptotic cells in pulmonary tissue at 0 h were 5% (Figure 7A). In oxygenated MEM, the apoptotic cells were 10% and 30% at 3 h and 6 h, respectively (Figure 7B–C). In unoxygenated MEM, the apoptotic cells were 10% and 20% at 3 h and 6 h, respectively (Figure 7E–F).

Additional file 1: Tables S1–S3 summarize the impacts of oxygen supply and anesthetic agents on the measured parameters; these results clearly demonstrate the need for tissue oxygenation and sevoflurane anesthesia.

## Discussion

The term cellular respiration implies delivery of O_2_ and metabolic fuels to the mitochondria, oxidation of reduced metabolic fuels with passage of electrons to O_2_, and ATP synthesis. Impaired respiration thus entails an interference with any of these processes. O_2_ consumption is a highly sensitive parameter in detecting cellular injury, since an intact biological system is required for consuming O_2_ and producing ATP. Interruptions at any level, such as the cell membrane, mitochondria and enzymes lead to impaired energy conversion and ATP production [4]. For example, mitochondria isolated from hamster lungs were inhibited by amiodarone (an antidysrhythmic agent with potent pulmonary toxicities) [9]. Similarly, inefficient oxidative phosphorylation was reported in lung mitochondria after exposure to environmental pollutants, such as dioxins and furans [10].

Cells are expected to repair damages if sufficient ATP is present. ATP depletion, on the other hand, inevitably leads to cell death. Thus, the fate of the cell, for most part, is determined by its bioenergetic status [11]. This fact reflects the inherent dependency of our biologic system on high rates of aerobic metabolism. Cancer cells, on the other hand, are capable of surviving on anaerobic metabolism (commonly referred to as: “aerobic glycolysis” or Warburg effect) [12].

One study suggested that the mitochondrial electron transport chain could be responsible for indirect DNA damages in lung tissue [13]. Therefore, monitoring cellular O_2_ consumption by mitochondrial cytochrome oxidase is important for assessing cytotoxicity.

The main purpose of this work was to develop an *in vitro* method to study the impact of respiratory pathogens and toxins on metabolic biomarkers (cellular respiration, ATP content and caspase activity) in lung tissue. The procedure described in Figure 1 resulted in reasonable preservation of lung tissue bioenergetics and structure (Figure 6B–C) for up to 6 h with minimum caspase activation. The success of this method relied on three critical procedural steps. The first step involved anesthesia and tissue collection. Anesthesia with sevoflurane and rapid (within 20 sec) removal of thin lung fragments from the animal were essential. It was vital to avoid organ ischemia or hypoxia before specimen collection, i.e., the organ should be well perfused during specimens’ collection. The second step involved tissue processing which required immediate immersion of collected tissues in *oxygenated ice-cold* MEM or KH buffer. The specimens were then cut into small slices using a sharp blade or punch biopsy device, while remaining in ice cold buffer or MEM continuously gassed with 95% O_2_:5% CO_2_. The third step involved sample incubation in a large volume solution (e.g., 50 mL) with continuous gassing with 95% O_2_: 5% CO_2_. Having adhered to these procedural steps, a coefficient of variation (Cv) for rates of respiration of 13% and for ATP of 53% over 6 h was achieved. The larger variation in ATP was likely reflecting inability to maintain a tissue perfusion comparable to that of *in vivo* and induction of apoptosis (Figure 7B–C).

Compared to unoxygenated MEM, a better-preserved lung structure was evident in oxygenated medium, despite time-dependent increases in apoptotic cells in both conditions (Figures 6, 7).

As noted in Figure 2, in unoxygenated solutions cellular ATP was depleted and lung structure was altered (Figure 6E–F), while rates of respiration were progressively increasing indicating uncoupling oxidative phosphorylation. This presumption was supported by the fact that caspase activity was noted by 2 hr (also see Figure 7E–F). Activation of caspases leads to opening of the transition permeability pores in the inner mitochondrial membranes, which results in uncoupling oxidative phosphorylation. This process results in rapid cellular ATP and nutrient depletions. These two consequences contribute to further damage of lung tissue. Thus, initial toxic effects become compound by overwhelming insults resulting from mitochondrial and metabolic derangements. At this stage, inevitable cell death occurs despite the removal of the initial toxic insult, as cellular repair requires ATP. These concepts are relevant to human lung diseases, such as adult respiratory distress syndrome (ARDS). Interestingly, the findings in Figure 2 were not reproduced in later experiments when lung tissue was incubated in O_2_ depleted buffer for 30 min (Figure 3). This result could be explained by residual cellular ATP under our experimental conditions (rather than its total depletion), and hence the tissue was able to endure this brief hypoxia.

In one study, the susceptibility of mitochondria isolated from pneumatocytes of pigs to *in vitro* hypoxia was investigated [14]. The results showed oxidative phosphorylation was unaffected by a 45-min hypoxia (as shown in Figure 3), suggesting the mitochondria were not directly responsible for damages observed after *in vivo* ischemia. Mitochondrial injuries were presumed to result from cellular insults, such as execution of apoptosis.

Urethane is a commonly used anesthetic agent in animal research. It is typically administered intraperitoneally. The results show that more caspases were induced in urethane compared to sevoflurane (Figure 4). Therefore, anesthesia with sevoflurane is recommended.

The mitochondrial function is best assessed in intact cells and tissues (rather than isolated organelles), using accurate determinations of cellular oxygen consumption and ATP synthesis. The conventional polarographic method for detecting oxygen is limited, especially for biological samples that require measurements over several hours [15]. The more recent use of fluorescence-based oxygen probes has significantly improved the detection methodology [6-8].

The results here are consistent with observed energy deficits during hypoxia in kidney proximal tubule cells [16]. Moreover, ischemia is well known to induce a mitochondrial damage that predisposes to superoxide anion production at the level of the respiratory chain [17]. For example, nitric oxide donors to the mitochondria (mediated by S-nitrosates thiol proteins) during ischemia inhibit O_2_ consumption [18]. Abnormal lung bioenergetics has been also reported in smokers (especially in patients with chronic obstructive lung disease and down-regulation of prohibitin-1) and with exposures to particulate matters of aerodynamic diameters of ≤10 μm [19,20].

As a proof of concept, Additional file 1: Figure S1 shows induction of caspases in the presence of 8 μM dactinomycin, a well known potent inducer of apoptosis [11]. Additional file 1: Figure S3 also shows the feasibility of utilizing the described method to monitor the effects of influenza A virus on cellular respiration and caspase induction following *in vitro* exposure of lung tissue to the virus.

The main limitation of the method described here is the need to collect and maintain viable lung tissue, avoiding ischemia and hypoxia. The lung should be well perfused and oxygenated throughout the procedure. Another limitation is the use of high oxygen concentration during *in vitro* incubation, which may promote free radical reactions. Thus, the described system is not ideal for testing pathogens, as exposure to high concentrations of O_2_ may produce non-physiological aberrations. The use of 95% oxygen, however, was not associated with noticeable toxicities (Figure 1). Nevertheless, this method may not be applicable to toxins that are likely to generate reactive oxygen species. Adequate buffering with a free radical scavenger (e.g., glutathione) may be helpful. The use of air-saturated buffer (Figure 2), intermittently oxygenated solution (Additional file 1: Figure S1, Panels A–C) or room air saturated with 5% CO_2_ (Additional file 1: Figure S2, Panel A) was associated with impaired cellular bioenergetics.

## Conclusions

The described method is relatively simple and requires minimal tissue handling. It permits comprehensive analyses (e.g., cellular respiration, ATP and caspase activity) over several hours. These biomarkers can be used to study the effects of toxins and pathogens on lung tissue.

## Competing interests

The author(s) declare that they have no competing interests.

## Authors’ contributions

ARA, MTA, and ASA designed the study, carried out the analysis, interpreted the data and drafted the manuscript. ARO measured caspase activity. SA and AA performed the histology. TP conceived of the study, measured respiration and ATP content. SMH and SMV prepared influenza virus. AKS supervised the progress and critically revised the manuscript. All authors read and approved the final manuscript.

## Supplementary Material

Additional file 1 **Figure S1.** Lung tissue respiration and caspase activity with and without 8 μM dactinomycin. **Figure S2.** Lung tissue respiration, ATP content and caspase activity at 5% CO_2_. **Figure S3.** Respiration and caspase activity in lung tissue exposed *in vitro* to influenza A virus (IAV). **Table S1.** Lung tissue respiration and ATP content in Wistar rats – Impact of continuous oxygenation. **Table S2.** Lung tissue caspase activity in Wistar rats – Impact of anesthesia. **Table S3.** Lung tissue caspase activity in C57Bl/6 and BALB/c mice (oxygenated buffer - sevoflurane inhalation). Click here for file
