# Supplementary material for: Lung tissue bioenergetics and caspase activity in rodents
Source: BMC Res Notes. 2013 Jan 12;6:12. doi: 10.1186/1756-0500-6-12 (PMC3557141; doi:10.1186/1756-0500-6-12)
Supplement: Additional file 1 — Figure S1. Lung tissue respiration and caspase activity with and without 8 μM dactinomycin. Figure S2. Lung tissue respiration, ATP content and caspase activity at 5% CO2. Figure S3. Respiration and caspase activity in lung tissue exposed in vitro to influenza A virus (IAV). Table S1. Lung tissue respiration and ATP content in Wistar rats – Impact of continuous oxygenation. Table S2. Lung tissue caspase activity in Wistar rats – Impact of anesthesia. Table S3. Lung tissue caspase activity in C57Bl/6 and BALB/c mice (oxygenated buffer - sevoflurane inhalation). [file 1756-0500-6-12-S1.doc]

**SUPPLEMENTARY MATERIALS**

**Lung Tissue Bioenergetics and Caspase Activity in Rodents**

Ahmed R. Alsuwaidi^1^; Mohammed T. Alsamri^1^; Ali S. Alfazari^2^;

Saeeda Almarzooqi^3^; Alia Albawardi^3^; Aws R. Othman^1^; Thachillath Pramathan^1^;

Stacey M. Hartwig^4^; Steven M. Varga^4^; Abdul-Kader Souid^1,*^

Departments of Pediatrics^1^, Medicine^2^ and Pathology^3^, United Arab Emirates University, P.O. Box 17666, Al Ain, UAE

Department of Microbiology^4^, Department of Pathology and Interdisciplinary Graduate Program in Immunology^5^, University of Iowa, Iowa City, IA 52242

(ARA): alsuwaidia@uaeu.ac.ae

(MTA): mohammed.alsamri@uaeu.ac.ae

(ASA): a.almelaih@uaeu.ac.ae

(SA): saeeda.almarzooqi@uaeu.ac.ae

(AA): alia.albawardi@uaeu.ac.ae

(ARO): aws.rashad@uaeu.ac.ae

(TP): pramathant@uaeu.ac.ae

(SMH): stacey-hartwig@uiowa.edu

(SMV): steven-varga@uiowa.edu

(AKS): asouid@uaeu.ac.ae

* To whom correspondence should be addressed: (AKS) e-mail: asouid@uaeu.ac.ae; Tel. +971-3-713-7429, fax +971-3-767-2022

**Figure S**1

| **A** | **B** |
| --- | --- |
| **C** | **D** |

**Figure S1. Lung tissue respiration and caspase activity with and without 8 μM dactinomycin.**  **Panels A-C:** Lung fragments from a C57Bl/6 mouse were incubated at 37^o^C with and without 8 µM dactinomycin in 50 mL KH buffer gassed with 95% O_2_:5% CO_2_ for 60 sec every hour. At indicated time points, samples were removed from the incubation medium and processed for measurements of O_2_ consumption and caspase activity. U (untreated), without dactinomycin; T (treated), with dactinomycin. **Panel A:** Runs of cellular mitochondrial O_2_ consumption are shown; *t* = 0 corresponds to animal sacrifice. The rate of respiration (*k,* μM O_2_ min^-1^) was set as the negative of the slope of [O_2_] *vs.* *t*. The values of *k_c_* (μM O_2_ min^-1^ mg^-1^) are shown at the top of the runs. **Panel B:** HPLC runs of caspase activity at 0 h, at 2 h without dactinomycin and at 2 h with dactinomycin. The running solvent was HPLC-grade methanol:dH_2_O 1:1 (isocratic). The retention time (*R*_t_) for Ac-DEVD-AMC was ~2.5 min and AMC ~4.8 min (insert, the AMC peaks, reflecting caspase activity). **Panel C:** The values of *k_c_* and AMC peak areas (÷10^6^) are plotted as a function of time of incubation. **Panel D:** Lung fragments from a C57Bl/6 mouse were incubated at 37^o^C with and without 8 µM dactinomycin in 50 mL KH buffer continuously gassed with 95% O_2_:5% CO_2_ for 4 h. Solvent A was HPLC-grade CH_3_CN:H_2_O [1:3, v/v] and Solvent B was dH_2_O (isocratic). The *R*_t_ for Ac-DEVD-AMC was ~2.5 min and AMC ~29.0 min.

**Figure S2**

| **A** | **B** |
| --- | --- |
| **C** | **D** |

**Figure S2 Lung tissue respiration, ATP content and caspase activity at 5% CO_2_.**  Lung fragments from a Wistar rat were incubated at 37^o^C in 50 mL MEM in room air saturated with 5% CO_2_ (in a CO_2_ tissue culture incubator). At indicated time points, samples were removed from the incubation medium and processed for measurements of O_2_ consumption, ATP content and caspase activity. **Panel A:** Runs of cellular mitochondrial O_2_ consumption are shown; *t* = 0 corresponds to animal sacrifice. The values of *k_c_* (μM O_2_ min^-1^ mg^-1^) and ATP content (pmol mg^-1^) are shown at the top of the runs. **Panels B-C:** HPLC runs of caspase activity at 0, 3, and 6 h with (Panel C) and without (Panel B) the addition of zVAD. The running solvent was HPLC-grade methanol:dH_2_O 1:1 (isocratic). The *R*_t_ for AMC peak was ~4.8 min. **Panel D:** The values of AMC peak areas corrected by sample weight (arbitrary unit mg^-1^ ÷10^6^) are plotted as a function of time of incubation.

**Figure S3**

| **A** | **B** |
| --- | --- |
| **C** | **D** |
| **E** | |

**Figure S3** **Respiration and caspase activity in lung tissue exposed *in vitro* to** **influenza A virus (IAV).** Lung fragments from a Wistar rat were incubated at 37^o^C in 50 mL MEM continuously gassed with 95% O_2_:5% CO_2_ with and without added 10 μL of the influenza A virus (IAV) suspension (IAV/PR8/34 10^8.1^ TCIU_50_). At indicated time points, samples were removed from the incubation medium and processed for measurements of O_2_ consumption and caspase activity. **Panel A:** Runs of cellular mitochondrial O_2_ consumption are shown; *t* = 0 corresponds to animal sacrifice. The values of *k_c_* (μM O_2_ min^-1^ mg^-1^) are shown at the top of the runs. **Panel B:** The values are *k_c_* are plotted as a function of time. **Panel C-D:** HPLC runs of caspase activity at 0, 3, 6, and 9 h without addition (Panel C) and with the addition of IAV (Panel D). The running solvent was HPLC-grade methanol:dH_2_O 1:1 (isocratic). The retention time (*R*_t_) for AMC was ~4.8 min. **Panel E:** The values of AMC peak areas (arbitrary unit mg^-1^ ÷10^6^) are plotted as a function of time of incubation.

**Table S1. Lung tissue respiration and ATP content in Wistar rats – Impact of continuous oxygenation**

| **Conditions** | ***k_c_***  **(μM O_2_ min^-1^ mg^-1^)** | **ATP content**  **(pmol mg^-1^)** |
| --- | --- | --- |
| Oxygenated buffer  Sevoflurane inhalation | 0.15 ± 0.04 (7)  CV = 26% | 91.0 ± 25.3 (6)  CV = 28% |
| Unoxygenated buffer  Sevoflurane inhalation | 0.12 ± 0.04 (6)  CV = 33% | 2.9 ± 1.4 (8) ^*^  CV = 48% |

Values are mean ± SD (n) for *t* <8 h. CV, coefficient of variation; * *p*<0.05

**Table S2. Lung tissue caspase activity in Wistar rats – Impact of anesthesia**

| **Conditions** | **Experiments** | **AMC peak area**  **(arbitrary units÷10^6^ mg^-1^)** |
| --- | --- | --- |
| Urethane inhalation | I | 46.5 ± 0.7 (5)  CV = 2% |
| Sevoflurane anesthesia | II | 2.1 ± 1.5 (4) ^*^  CV = 71% |
|  | III | 2.7 ± 1.6 (4) ^*^  CV = 59% |
|  | IV | 2.6 ± 2.1 (8) ^*^  CV = 81% |

Values are mean ± SD (n) for *t* <8 h. CV, coefficient of variation; * *p*<0.05

**Table S3 Lung tissue caspase activity in C57Bl/6 and BALB/c mice (oxygenated buffer - sevoflurane inhalation)**

| **Experiments** | ***k_c_***  **(μM O_2_ min^-1^ mg^-1^)** | **AMC peak area**  **(arbitrary units÷10^6^ mg^-1^)** |
| --- | --- | --- |
| **C57Bl/6 mice** | | |
| I | 0.16 ± 0.02 (4)  CV = 13% | 0.13 ± 1.3 (5) |
| II | 0.15 ± 0.05 (4)  CV = 33% | 3.3 ± 2.5 (4)  CV = 76% |
| **BALB/c mice** | | |
| 8 independent experiments | 0.08 ± 0.03 (11)  CV = 38% | - |

Values are mean ± SD (n = number of runs for *t* <8 h). CV, coefficient of variation
